# A controlled trial of automated classification of negation from clinical notes

**DOI:** 10.1186/1472-6947-5-13

**Published:** 2005-05-05

**Authors:** Peter L Elkin, Steven H Brown, Brent A Bauer, Casey S Husser, William Carruth, Larry R Bergstrom, Dietlind L Wahner-Roedler

**Affiliations:** 1 Department of Medicine, Mayo Foundation, Rochester, MN, USA; 2 Department of Biomedical Informatics Vanderbilt University, Nashville, TN and the Veterans Health Administration (VHA), USA; 3 Department of Internal Medicine, Johns Hopkins School of Medicine, Baltimore, MD, USA

## Abstract

**Background:**

Identification of negation in electronic health records is essential if we are to understand the computable meaning of the records: Our objective is to compare the accuracy of an automated mechanism for assignment of Negation to clinical concepts within a compositional expression with Human Assigned Negation. Also to perform a failure analysis to identify the causes of poorly identified negation (i.e. Missed Conceptual Representation, Inaccurate Conceptual Representation, Missed Negation, Inaccurate identification of Negation).

**Methods:**

41 Clinical Documents (Medical Evaluations; sometimes outside of Mayo these are referred to as History and Physical Examinations) were parsed using the Mayo Vocabulary Server Parsing Engine. SNOMED-CT™ was used to provide concept coverage for the clinical concepts in the record. These records resulted in identification of Concepts and textual clues to Negation. These records were reviewed by an independent medical terminologist, and the results were tallied in a spreadsheet. Where questions on the review arose Internal Medicine Faculty were employed to make a final determination.

**Results:**

SNOMED-CT was used to provide concept coverage of the 14,792 Concepts in 41 Health Records from John's Hopkins University. Of these, 1,823 Concepts were identified as negative by Human review. The sensitivity (Recall) of the assignment of negation was 97.2% (p < 0.001, Pearson Chi-Square test; when compared to a coin flip). The specificity of assignment of negation was 98.8%. The positive likelihood ratio of the negation was 81. The positive predictive value (Precision) was 91.2%

**Conclusion:**

Automated assignment of negation to concepts identified in health records based on review of the text is feasible and practical. Lexical assignment of negation is a good test of true Negativity as judged by the high sensitivity, specificity and positive likelihood ratio of the test. SNOMED-CT had overall coverage of 88.7% of the concepts being negated.

## Background

A great wealth of patient specific medical data is stored as transcribed free text. While this format is useful for individuals reading the medical record, information stored as free-text is difficult to use in decision support systems or automated cross population studies [1]. Efforts to extract computer-usable information from free text archives vary widely. Traditionally, teams of trained abstractors have manually reviewed patients' charts. String matching is a simple algorithmic approach. Identifying concepts is a much more complex process. Algorithmic natural language understanding holds great promise, but remains difficult to achieve [2,3]. Despite the challenges, a number of groups have applied natural language processing techniques with varying degrees of success [4-10]. Concept-based indexing is another approach that has been applied to a number of areas including literature retrieval, health related web sites, clinical diagnoses, and medical narratives [11-16].

Natural language processing is routed in a logical representation of discourse. Until the 1920s logic and mathematics was considered spiritual not scientific. Since the time of Pythagoras, mathematics was considered a revelation of the divine order. In Principia Mathematica (Russell and Whitehead), demonstrated that mathematics was logical. Logical positivism was then applied to science and psychology.

Noam Chomsky's seminal work "The Logic Structure of Linguistic Theory," was published in 1955 in mimeograph form and in press in 1975. This work expressed the view that language was a cognitive activity and required a meta-model of language to effectively communicate. He demonstrated that the stimulus response model could not account for human language. This idea that language is processed led to the application of computer science to free text (natural language) processing. Computational linguistics (CL) is a field of computer science which seeks to understand and represent language in an interoperable set of semantics. CL overlaps with the field of Artificial Intelligence and has been often applied to machine translation from one human language to another. Naomi Sager in 1994 published in JAMIA a paper entitled "Natural Language Processing and the Representation of Clinical Data." Here Dr. Sager showed that for a set of discharge letters a recall of 92.5% and a precision of 98.6% could be achieved for a limited set of pre-selected data using the parser produced by the Linguistic String Project at New York University [1-3].

In 2004, Friedman et al reported a method for encoding concepts from health records using the UMLS [4]. In this study Dr. Friedman and colleagues used MedLEE to abstract concepts from the record and reported a recall of 77% and a precision of 89%. In 2001, Nadkarni provided a description of the fundamental building blocks needed for NLP [5]. He discussed their method for lexical matching and part of speech tagging in discharge summaries and surgical notes. Henry Lowe developed MicroMeSH an early MUMPS based terminology browser which incorporated robust lexical matching routines. Dr. Lowe working with Bill Hersh reported the accuracy of parsing radiology reports using the Sapphire indexing system [6]. Here they reported good sensitivity and they were able to improve performance by limiting the UMLS source vocabularies by section of the report.

MetaMap has the capacity to be used to code free text (natural language) to a controlled representation which can be any subset of the UMLS knowledge sources [7]. MetaMap uses a five step process which begins by using the SPECIALIST minimal commitment parser which identifies noun phrases without modifiers. The next step involves the identification of phrase variants. These variants are then used to identify candidate phrases from within the source material [8]. Linguistic principals are used to calculate a score for each potential match. Brennan and Aronson used MetaMap to improve consumer health information retrieval for patient [9].

We have built and described systems for concept based indexing, automated term composition, and automated term decomposition. In its current version, the system uses the SNOMED-CT terminology. The accuracy of this automated technique has previously been evaluated [10]. Many individuals have evaluated the accuracy of manual term composition [11,12]. The clinical coding center of the NHS has reported limited success with their own algorithm for automated term dissection in the past [13,14].

As we move toward compositional terminologies, the need to organize the terms within a compositional expression becomes important for both the readability and understanding of these composite terms [15,17]. Identifying concepts that are explicitly asserted as not being the case and separating them from positive assertions becomes of critical importance if we are to understand the implications of medical text. Linguistic negation is a challenging problem [18]. This trial evaluates a mechanism for automated assignment of negation status to concepts parsed from the terminology using a negation ontology. The text is analyzed to identify expressions indicating negation and a model of negation is applied to assign values to concepts. We have named this system the automated negation assignment grammar [10]. We recognize the following semantic types: Kernel concepts, Modifiers, Qualifiers or Negative Qualifiers [19]. A rule base is then applied which organizes the Modifiers, Qualifiers and Negative Qualifiers around the Kernel concepts. These are represented in a hierarchical structure with the degree of indentation being representative of semantic dependency. The accuracy of this automated technique has previously been evaluated [10]. Many individuals have evaluated the accuracy of manual term composition [11,12].

Identifying concepts that are explicitly asserted negatively (e.g. "no evidence of pneumonia") and separating them from positive assertions becomes of critical importance if we are to understand the implications of medical text.

To illustrate the importance of concept negation, we reference a case of a 62 year old female who presents with erythema over the dorsum of the left foot with exquisite tenderness over a wound situated over the mid foot. After a comprehensive clinical work up, she was found to have a Cellulitis of the left foot without signs of lymphangitic spread of her infection. In this case, it is an important distinction that our patient did not have "Lymphangitis" associated with her "Cellulitis, left foot," as opposed to a distinct separate case where the diagnosis of "Lymphangitis was present." Epidemiologically, if one was studying Lymphangitis, it would be important to exclude this patient's record from the analysis.

A previous study of Negation by Mutalik et al, described the lexical assignment of negation using the UMLS to code free text documents. Their intervention had a sensitivity of 95.7% and a specificity of 91.8% [20]. They did not report the UMLS coverage of the concepts that appeared in the text. They also noted that the words "no", "not" "denied/denies" and "without" made up 92.5% of the negation in their study. Chapman et al looked to identify negation in discharge summaries and identified negative UMLS concepts with a sensitivity of 77.8% and a specificity of 94.5% using regular expressions [21].

## Methods

### Study Design

Forty-one unique clinical records (which were notes comprising a set of history and physical medical evaluations) of forty-one separate patients were randomly selected from the outpatient section of the Department of Internal Medicine at John's Hopkins Medical School. These were indexed using the Mayo Vocabulary Server. The records are presented to the system as free-text ASCII files. The text is parsed using the Mayo Health Record Parser, which parses the text into sections consistent with the usual health record as presented below:

History

    History of Present Illness

       By Problem

    Past Medical History

    Social History

    Medications

    Allergies

    Review of Systems

Physical Examination

    By Body Part

Diagnostic Testing

Assessment / Report / Plan

    By Problem

### Negation Assignment

**Figure 1:**
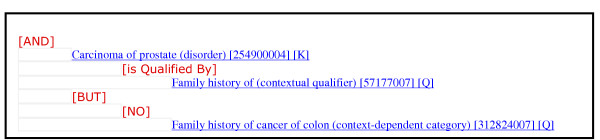
Fictitious example of the assignment of negation for the clinical statement "Mr. Jones has a family history of carcinoma of the prostate, but has no family history of colon cancer." The statement *"No" family history of cancer of colon *represents a negative assertion.

Negation is part of a larger system that assigns to concepts a level of certainty as part of the generation of a two-phase dyadic parse tree. Each sentence within each section is parsed first by a preprocessor, which breaks the input into text and operators (i.e. And, Or, Not, Maybe). The text is parsed using the Mayo Vocabulary Server, which returns a set of concepts representing the best match from within SNOMED-CT to the sentence fragment parsed. A rule base is applied to the text that assigns to each concept an attribute stating that the concept is a positive assertion, a negative assertion or an uncertain assertion. The software is not yet developed to a point where it could be used by other users, and thus is not freely available, but the authors will provide access to the software to any readers interested in validating the results. In this manuscript, we are focusing on the evaluation of the assignment to concepts the attribute "negative assertion" (see Figure 1). Mixed assertions such as "probably not", were considered uncertain assertions for the purposes of this evaluation.

The system uses the SNOMED-CT terminology to index clinical documents. The entire terminology was employed so that any SNOMED-CT concept appearing in these histories, regardless of their location within the ontology, would be codified. The text is analyzed to identify expressions indicating negation and a model of negation is applied to assign values to concepts. We call this the automated negation assignment grammar.

Examples of the types of terms which imply negation are "no", "denies", and has "ruled out." An example of terms, which stop the propagation of the assignment of negation, is "other than" (e.g. *The patient *denied **a history of previous cardiac disease **other than* palpitations which he experienced while giving a presentation resulting in syncope*.). In the previous example, positive assertions are in italics and negative assertions are in bold and the operators are underlined. The larger ontology of negation terms their lexical variants and the associated rules for applying them are being made available within a larger vocabulary server named the Mayo Vocabulary Server.

The results of the parsing were reviewed by an expert medical terminologist (independent from the study team). We chose to employ a single reviewer in this study who was independent from the study team, based on the high inter-rater agreement found in our recent evaluation of SNOMED-CT for the coding of problem list data (94.3% agreement; Kappa Statistic = 0.79) and our belief that the assignment of negation was relatively straightforward when compared with most health terminology judgments. For each occurrence of negation in the text as judged by a human reviewer, the number of correctly mapped negative concepts and the total number of negative concepts were tallied by subsection of the record. If the assertions were not tagged correctly, a record was made to distinguish whether there was a failure to map to the terminology, or if the engine simply mapped the term incorrectly. The failure analysis also included whether the missing information was a Kernel concept (the main point of the expression), a Modifier (a concept that changes the meaning of a term in a clinical sense like "severity"), a Qualifier (a concept that changes the meaning of a term in an administrative or temporal sense like "recurrent").

The sensitivity, specificity and positive predictive value and positive likelihood ratios for the assignment of the negation status are reported.

For an example of an in context negation assertion as found in a medical record, see figure 1.

### Statistical Analysis

In addition to descriptive comparisons of the accuracy rates for the assignment of negation, a formal statistical comparison was performed. To determine if the results we obtained could be accounted for by chance alone the following method was employed. Testing was employed for equality of accuracy rates between the negation assignment and a coin flip to determine if the effect, which we have seen, could have been present from chance alone by the Pearson Chi-square statistic for equality of proportions.

## Results

Overall, we identified 14,792 health concepts in the text of 41 clinical records using SNOMED-CT the Mayo Vocabulary Server Parsing software and the manual review. No attempt was made to filter out duplicate concepts. 13,082 of these were positive or uncertain assertions of which 12,921 were recognized by the parser as such. Out of the 2,028 negative concepts, 205 Concepts were not mapped by SNOMED-CT, but were identified by the human reviewer. Of the remaining 1,823 concepts the engine identified 1662 correctly (p < 0.001, Pearson Chi-Square test; when compared with a coin flip). One-hundred and sixty-one concepts were incorrectly assigned as negatives and another 48 were assigned incorrectly as positives. This resulted in the two-by-two table shown in Table 1. The sensitivity (Recall) of the assignment of negation was 97.2% (range 50% to 100%) and the specificity of that same assignment was 98.8% (range 33.33% to 100%). The positive likelihood ratio for the effect was 81, which indicates that our method is a good test for identifying negation. The reliability of this test as judged by the positive likelihood ratio compares favorably with other acceptable medical diagnostic tests such as the dobutamine stress echo that has a positive likelihood ratio of 24. The negative predictive value of the assignment was 99.6%. The positive predictive value (Precision) was 91.2%.

**Table 1:** Two by Two table of the results of the negation mapping compared with the initial human review. The "C" prior to the operator signifies Computer identification (e.g. Cnegative). True negation is based on the human review that served as our gold standard.

	**True Negation**	**True (Positive or Uncertain)**
**CNegative**	1662	161
**Cpositive Or Cuncertain**	48	12921

In Table 1 we show the results of the human review as compared with the automated assignment of Negation. As all concepts were coded as Positive, Uncertain or Negative assertions, we combined the Positive and Uncertain assertions for the purposes of this analysis. True negation was the rate of negation identified by the human reviewer from within the text. True positives or uncertain were the other concepts, which were encoded by the automated engine and tagged as either being any of the three types of assertions. "Cneg" is the computer-generated rate of assignment of negation and "Cpos" and "C?" are the rates of assignment of positivity or uncertainty.

The failure analysis showed that many of the concepts which were assigned as positive which should have been negative, were words such as "nontender" and "colorless" which were missed by our algorithm. Another class of problems stemmed from operators which appeared to the engine to be double negatives such as "but not" or "but it never."

In Table 2, the sensitivity and specificity of the negation routine varied throughout the different sections of the health record. Incalculable values were created when the true positives plus false negatives (TP + FN) were zero or when the true negatives plus the false positives (TN + FP) were zero (otherwise the equation for sensitivity or specificity would require division of the numerator by zero)

**Table 2:** The sensitivity and specificity of the negation routine by area of the record.

**section**	**subsection**	**avg_sens**	**avg_spec**
History	--	92.30	80.0
History	Allergies	100.0	Div 0
History	Current meds	Div 0	Div 0
History	Family history	89.19	57.14
History	HPI	94.71	64.44
History	Medications	50.0	100.0
History	PMH	94.12	10.0
History	Social history	98.91	66.67
History	Systems rev	99.43	88.89
Exam	abdomen	98.39	50.0
Exam	breasts	100.0	Div 0
Exam	ENT	96.52	33.33
Exam	Extremities	98.67	Div 0
Exam	eyes	95.0	50.0
Exam	gait	100.0	Div 0
Exam	genitalia	100.0	Div 0
Exam	head	Div 0	Div 0
Exam	heart	100.0	100.0
Exam	lungs	100.0	Div 0
Exam	lymph	100.0	Div 0
Exam	musculo	100.0	Div 0
Exam	neuro	100.0	Div 0
Exam	rectum	100.0	100.0
Exam	skin	100.0	Div 0
Exam	thyroid	100.0	Div 0
Exam	vitals	91.11	Div 0
Diagnostic Testing	--	100.0	100.0
Assessment	--	87.78	69.01
Assessment	Addendum	50.0	71.43
Assessment	Plan	70.0	Div 0
Assessment	Problem/diagnosis	64.71	66.67

## Discussion

The assignment of negation to concepts from a controlled terminology such as SNOMED-CT can be automatically assigned reliably. The assignment had a high positive likelihood ratio indicating that it is overall an accurate test of the records for this condition. The most common reason for failure was the inability of SNOMED-CT to represent the negative concepts. This was verified by browsing the terminology for the concept as well as by inability of the mapping engine to identify a correct match. The predictive value of the assignment of negation was highly statistically significant when compared with a coin flip (chance alone) with a p < 0.001.

The failure analysis identified unexpected methods of negation, which we are dealing with in a second generation of the software that handles roots and stems. We have also created an ontology of terms that start negation and another set which stop the propagation of the assignment of negation. Clearly there was variability in the accuracy of the algorithm across the various sections of the clinical record. This provides the Informatics research community with an opportunity to identify areas of focus for future research efforts.

We extend the work of Mutalik et al and Chapman et al by performing this study using SNOMED-CT and by utilizing a second independently developed ontology for negation. Also we used full medical evaluations in our study, which had a higher per case percentage of negative concepts as compared with the surgical reports and discharge summaries used in studies by previous authors. Previous unpublished usability data in our laboratory noted that clinicians require 95% accuracy for acceptance of a system that provides conceptual coding of clinical content. Therefore the negation algorithms should be an acceptable starting point for clinical applications for the physical examination, the HPI, social history, allergies and review of systems sections of the clinical record. More work is needed to understand the negation requirements of medications, family history, the vital signs, and the assessment section of the clinical record.

Compositional terminologies are one promising answer to the problem of clinical content completeness [22]. High-quality controlled health vocabularies provide a gateway to improved clinical data availability for outcomes research, utilization review, and improved management of the electronic medical record [23]. This promise is contingent upon data entry mechanisms, which will not disrupt the flow of a busy practice [24].

Creating well formed compositional expressions using a controlled health vocabulary can be labor intensive and time consuming. Given the ever-increasing demands on clinicians' time, we must work to create mechanisms, which aid the busy clinician as we migrate toward, an electronic clinical environment. Missed negation can lead to excess testing which in turn can lead to an increased rate of medical error. Likewise erroneous assignment of negation can lead to missing allergies and other important health data that can negatively impact patient safety. Automated tools designed to assist clinicians with the formulation of compositional expressions are necessary if we are to make use of powerful compositional terminologies.

## Competing interests

The author(s) declare that they have no competing interests.

## Authors' contributions

All authors worked on the design of the study and reviewed the manuscript. PE, BB and DWR supervised the actual running of the experiment. All authors participated in the analysis of the results.

## Pre-publication history

The pre-publication history for this paper can be accessed here:


